# Growth Performance, Nutrient Digestibility, and Retention in Atlantic Salmon, *Salmo salar* L., Fed Diets with Fermented Sugar Kelp, *Saccharina latissima*

**DOI:** 10.1155/2023/6664947

**Published:** 2023-11-29

**Authors:** Sahar Sartipiyarahmadi, Antony J. Prabhu Philip, Harald Sveier, Silje Steinsund, Erik-Jan Lock, Angelico Madaro, Tom Johnny Hansen, Martin Wiech, Øivind Strand, Jan Vidar Jakobsen, Marleen E. van der Heide, Jan Værum Nørgaard, Sofie C. Remø

**Affiliations:** ^1^Institute of Marine Research (IMR), Bergen, Norway; ^2^Department of Biological Sciences, University of Bergen, Bergen, Norway; ^3^Nutrition and Feed Technology Group, Nofima, Bergen, Norway; ^4^Lerøy Seafood Group ASA, Bergen, Norway; ^5^Cargill, Dirdal, Stavanger, Norway; ^6^Department of Animal and Veterinary Sciences, Aarhus University, Aarhus, Denmark

## Abstract

Using low trophic marine resources such as sugar kelp (*Saccharina latissimi*) is of great interest to increase the circular food production in the ocean. Sugar kelp does, however, contain high levels of carbohydrates and iodine and does not have considerable levels of protein and lipids, which may make it less suitable as a feeding ingredient. A 10-week feeding trial was done to investigate the effect of graded dietary inclusion levels of fermented sugar kelp (FSK), on growth performance, digestibility, retention of nutrients, and mineral composition in postsmolt Atlantic salmon (*Salmo salar* L.). The experimental diets were made to simulate a standard grower feed for salmon postsmolts in SW with ∼63% plant-based ingredients vs ∼34% marine ingredients and increasing concentrations of FSK between 0% (control feed) and 4% of the diet. During the feeding trial, the weight gain and specific growth rate (SGR) decreased linearly with increasing dietary FSK levels, where the SGR was slightly reduced from 1.2% for the fish given the control feed to 1.1% in the fish given feeds containing 3% and 4% FSK. This resulted in a lower weight gain of up to 9% in the fish given 4% FSK compared to the control. Feed intake and feed conversion ratio were, however, similar in all diet groups, and FSK inclusion did not influence the digestibility of macronutrients or minerals, except for lipid. The reduced growth is likely related to a lower digestible energy level in the diets, and the retention of both lipids and energy was affected by FSK inclusion. Inclusion of FSK also influenced iodine availability and retention, as well as increasing iodine status in whole body and muscle in a dose-dependent manner until reaching a plateau, which corresponds to 124 mg I kg^−1^ WW (135 mg I kg^−1^ DW), at 3% FSK inclusion.

## 1. Introduction

The aquaculture industry is rapidly expanding and is expected to continue growing worldwide. However, the industry faces a major challenge due to a shortage of available feed resources [1]. Therefore, it is crucial to explore the use of new alternatives for sustainable feed ingredients from underutilized and renewable natural resources that do not compete with human food [2], as well as improving processing technology to produce safe and nutritious aquafeed ingredients [3]. Low trophic species that are produced or cultivated are considered to have potential as future feed sources [3]. The aquaculture industry is increasingly looking toward marine macroalgae (seaweed) as a resource for use in feeds due to their high growth rate, potential cultivation in salt water, and no requirements for arable land or industrial fertilization [2, 4, 5]. In addition to that, macroalgae contribute to food circularity by taking up dissolved inorganic nutrient wastes from water [6, 7]. Macroalgae are known for their high nutritional quality and are a promising supplement in functional foods or a potential source for extracting compounds [8, 9]. They are a rich source of essential amino acids, beneficial polysaccharides, vitamins, minerals [9, 10], and bioactive substances [11].

Norway aims to produce 5 million metric tons (MT) of salmonids by 2050, which would require 6 million MT of feed [12]. Sugar kelp is one of the most cultivated macroalgae species in Europe and Norway [5, 13–15] with potential economic value as animal feed and food for human consumption [13]. In 2014, the first permission for sea cultivation of macroalgae was launched in Norway, and experience shows the potential in both monoculture and integrated multitrophic aquaculture system [16]. Cultivated macroalgae accounted for 97.1% of the world's annual production of macroalgae (including wild and cultivated) in 2018, which totaled 32.4 million tons [17]. However, the low crude protein (1%–21% of dry matter), low lipid content (0.5%–3.4% of dry matter), and high levels of complex carbohydrates, ash [18], and moisture content (75%–90%) [9, 19] in this species [9, 20, 21] pose challenges for its application in aquafeed.

Previous feeding studies have proven that overall performance of fish on macroalgae in the diet depends on the fish species (species specific) and inclusion level (dose dependent) of macroalgae [22–24]. As shown in Table 1, the incorporation of macroalgae in aquafeed at low levels (<10%) can maintain or enhance growth performance (weight gain, feed utilization, and survival). However, fish growth and feed efficiency might be negatively affected at high inclusion level (≥10%) of macroalgae due to the presence of antinutritional factors such as lectins, protease inhibitors, tannins, phytate, and toxins which are widely distributed in plants and macroalgae [25–27] and low level of energy content [2]. In rainbow trout (*Oncorhynchus mykiss*) including up to 2% dried sugar kelp in the diet did not reduce growth or feed utilization, whereas both weight gain and specific growth rate (SGR) were reduced when including 4%, likely due to decreased protein digestibility [28]. Hence, a critical aspect when developing diets for fish is the evaluation of their capacity to digest novel ingredients and determining the appropriate inclusion level in addition to optimizing feed use. Optimal feed utilization is important to reduce feed costs and environmental impacts such as greenhouse gas emission [29, 30].

Brown macroalgae such as sugar kelp are known as iodine-rich sources containing up to 10,000 mg iodine kg^−1^ dry weight (DW) [31, 32]. However, concerns have been raised about using high levels of sugar kelp in the diet of Atlantic salmon.

To achieve large-scale use of macroalgae as a feed resource, it is crucial to address the challenge of a steady supply of biomass. Seasonal harvesting necessitates proper processing, preservation, and long-term storage [2]. Fermentation is a promising preservation method for brown macroalgae [33] and commonly uses lactic acid bacteria (LAB) [34]. Fermentation is a simple and cheap method for stabilizing a wet biomass that would otherwise rapidly degrade after harvesting [35]. Furthermore, it enhances the shelf life, food safety, and nutritional and sensory properties of the product [34, 36]. Fermentation also affects the nutrient profile and protein digestibility of macroalgae [2]. It lowers crude fiber content and increases protein digestibility, thereby improving its nutritive value as fish feed [2, 37]. Fermentation also reduces the high content of iodine in macroalgae [38]. However, the low levels of DM content of macroalgae species must be considered as a challenge for incorporating them into the diet and pelletizing.

There are few available publications on the inclusion of sugar kelp or other brown macroalgae in feed for fish particularly salmonids as one of the most important groups of aquaculture fish species. Moreover, the generation of novel feed products by fermentation technology has yet only been developed for a few macroalgae species, particularly red algae [38, 39]. Therefore, this study investigated whether including fermented sugar kelp (FSK) (1%, 2%, 3%, and 4%) in Atlantic salmon diet influences growth performance, nutrient digestibility and retention, whole body and muscle composition, and welfare of fish.

## 2. Materials and Methods

### 2.1. Ethical Statement

The feeding trial was conducted at Matre Research Station, Norway, according to the Norwegian regulations on animal experimentation. The experimental protocol was approved by the Norwegian Food Safety Authority (FOTS ID # 25202).

### 2.2. Fermented Sugar Kelp

The FSK was provided by Ocean Forest AS, Lerøy Seafood Group. The sugar kelp was cultivated and harvested by Ocean Forest AS at Trollsøy, Austevoll (Norway; 60° 7.821′, 5° 14.891′) in May 2020. The fermentation process was initiated immediately after harvest on fresh material at ambient temperature (8–14°C) in closed intermediate bulk containers, by adding 10 g of a commercial blend of *Lactobacillus* bacteria (LAB) delivered by European Protein (Pig Stabilizer 600, Version 04.12.2017) per 1,000 kg of finely chopped sugar kelp. The pH dropped to below 4.0 within a span of 3 weeks, which was sustained thereafter. The composition of both fresh sugar kelp, sampled prior to the fermentation process, as well as the FSK is presented in Table 2. Both fresh and FSK contained 1.3 g 100 g^−1^ WW (15% DW) crude protein and less than 1% g 100 g^−1^ WW lipid content, while FSK contained somewhat less carbohydrate than fresh sugar kelp. The cellulose level was similar in both fresh and FSK. Both groups showed the same concentration of manganese (Mn) and zinc (Zn), while copper (Cu), selenium (Se), iron (Fe), and iodine levels were different after fermentation.

### 2.3. Experimental Diets

The feeding trial was designed as a dose–response study using graded inclusion levels of FSK. The control diet was formulated as a commercially relevant reference feed for postsmolt in seawater. In the experimental diets, FSK was added to reach the target levels of 1%–4% in the finished extruded pellets (Table 3). All diets were formulated to meet the minimum requirements of Atlantic salmon [40]. In the finished diets, the protein level ranged between 43 and 46 g 100 g^−1^ WW (Table 3). The amino acid profile in the experimental diets was still comparable to the experimental diets (Table 4). The FSK incorporation in the diet resulted in lower lipid (25–18 g 100 g^−1^ WW) and lower digestible energy (DE) (19–18 MJ kg^−1^ WW) in FSK4% diet compared with the control group. Diets with a higher FSK contained lower NDF and hemicellulose content, while the others were comparable between the experimental diets. Some variations were seen in dietary Fe and Se levels and iodine ranged between 4 and 138 mg kg^−1^ WW in the experimental diets. The experimental diets were produced by Cargill (Dirdal, Norway). To determine apparent digestibility/availability of nutrients, yttrium oxide (0.02% ≈ 200 mg/kg) was added as an inert marker to all diets.

### 2.4. Fish and Rearing Condition

At the start of the experiment, 65 Atlantic salmon (*Salmo salar* L.) postsmolts with an average weight of 204 ± 37 g (mean ± SD) were randomly distributed in 15 quadrangular 1.5 m^3^ glass fiber tanks, in total 975 fish, and the five experimental diets were each randomly assigned to triplicate tanks. The postsmolt used in the present study originated from Aqua Gen strain, Agua Gen AS, Trondheim, Norway. In each tank, 55 fish were produced from commercially available eggs obtained in the fall of 2019 (mixed population) and 10 fish were from an isogenic salmon line (all-male population) produced at Matre, also originally made from the Aqua Gen strain in 2011 [41, 42]. The all-male fish were included as a standard reference fish to eliminate the influence of genetic variation on the growth evaluation in the study, and these were pit tagged for determination of individual growth rates. The fish were acclimatized to the tanks for 3 weeks prior to experimental start. The average density of each tank at the start of the experiment was 10.0 ± 0.5 kg m^−3^ (mean ± SD).

During the experiment, the environmental conditions were kept within normal production regimes for Atlantic salmon postsmolt. The fish were kept in seawater with a salinity of 34 ppt that was provided using a flow-through system, and the water flow was adjusted as the fish grew to maintain oxygen saturation in the tanks. The water temperature ranged between 8.8 and 9.2°C with a mean of 9 ± 0.07°C (mean ± SD) during the experimental period, under continuous (24 hr) light.

The fish were given two meals per day (between 9 : 30 to 11 : 00 and 12 : 30 to 14 : 00) for 10 weeks. The fish were fed in excess with automatic feeders (Arvotec TD 2000) to ensure enough feed for all the fish, and the feeding rate was adjusted according to the increase in fish biomass. The uneaten feed pellets were collected 15min after each meal to estimate feed intake according to Helland et al. [43].

### 2.5. Sampling Procedure

All sampled fish were euthanized with an overdose of tricaine methane sulfonate (500 mg/L, FINQUEL MS-222). At the start of the experiment, 45 fish (30 fish from the mixed population and 15 fish from all-male population) were sampled to register organ weights (viscera, liver, and heart), as well as determination of organ-specific nutrient compositions. The same number of fish (*n* = 45) were sampled to determine the whole-body proximate composition and were divided into three pools each (*n* = 30 fish from the mixed population, *n* = 3 pooled, and *n* = 15 fish from the all-male population, *n* = 3 pooled). At the end of the experiment, weight and length were recorded on all fish. From each tank, 10 fish from mixed population and 10 fish from all-male population were sampled for determination of whole-body and organ-specific nutrient compositions, where five whole fish from each were pooled for determination of whole-body composition (*n* = 5 fish per tank, *n* = 3 per diet, pooled) and five fish were dissected individually for registrations of viscera, liver, and heart to calculate somatic indices (*n* = 5 fish per tank, *n* = 15 per diet). The whole fish, as well as the whole muscle, were frozen in dry ice, homogenized, and stored at −20°C for determination of nutrient composition (*n* = 5 fish per tank, *n* = 3 per diet, pooled).

A visual evaluation was done on the 20 individuals sampled from each tank (*n* = 20 fish per tank, *n* = 60 per diet) prior to dissection to monitor standard welfare indicators and operational indicators, including eye status, jaw wound and deformity, opercula status, spine deformation, gill condition, condition factor, and skin and fin damage according to a standard scoring system (SWIM) [44, 45]. Cataract examination was performed in darkened conditions using a Heine HSL 150 hand-held slit lamp (HEINE Optotechnik GmbH & Co. KG, Herrsching, Germany) [46]. Cataracts were graded 0–4 on each lens, according to the criteria given by Wall and Bjerkas [46].

Feces were collected by stripping (gently expelled using light pressure on the abdomen near the vent) from 55 fish per tank (45 fish from mixed population and 10 fish from all-male population) at the end of the trial, and feces of each population (all-male and mixed) were separately pooled for a composite sample used to determine the apparent digestibility (ADC)/availability coefficient (AAC) of nutrients (*n* = 45 fish from the mixed population per tank, *n* = 3 per diet, pooled and *n* = 10 fish from the all-male population per tank, *n* = 3 per diet, pooled).

### 2.6. Analytical Methods

DM, crude protein, crude fat, ash, gross energy, and carbohydrate content were determined in the raw materials (fresh and FSK), experimental diets, whole body, and feces samples. Briefly, DM was measured after drying to constant weight at 105°C for 24 hr [47]. Crude protein was analyzed using a protein analyzer (Vario Macro Cube, Elementar Analysen Systeme GmbH, Germany) [48]. Crude fat of the feed, tissue, and feces samples was extracted with ethyl acetate and filtered before the solvent evaporated and the fat residue was weighed. The method is standardized as a Norwegian Standard, NS 9402 [49]. Crude fat of the raw material samples was also measured based on the gravimetry after acid hydrolysis [50]. Combustion in a muffle furnace at 550°C for 16–18 hr determined ash content, and gross energy was measured using an IKA calorimeter C7000 after drying the homogenized diet samples for 48 hr at 60°C. To determine total nonstarch polysaccharides (T-NSP) and their constituent sugars gas–liquid chromatography was used for neutral sugars, and colorimetry was used for uronic acid as modified and described by Englyst, Wiggins et al. [51], Englyst, Quigley et al. [52], Theander, Åman et al. [53], and Knudsen [54]. Total NSP contains cellulose and soluble and insoluble noncellulosic polysaccharides (NCP) based on the analysis of monomeric constituents. Cellulose was determined as the difference of glucose content of NSP when the swelling step with 12 M H_2_SO_4_ was included (NSP_Glucose (12 M H2SO4)_) or omitted (NSP_Glucose (2 M H2SO4)_). The sum of glucose, galactose, xylose, arabinose, mannose, rhamnose, fucose, and uronic acids shows T-NCP. Insoluble residue after hydrolysis with 12 M H_2_SO_4_ determined the lignin-like substances. The fractions in macroalgae that were insoluble in sulfuric acid and consequently indigestible and not fermentable were recognized as lignin. However, it could not be determined whether it is lignin or other acid-insoluble components in macroalgae the fraction will be referred to as the lignin-like substance. The sum of lignin-like substances and T-NSP corresponds to total dietary fiber (T-DF). Neutral detergent fiber (NDF), acid detergent fiber (ADF), acid detergent lignin (ADL), hemicellulose, and cellulose were measured in the feed and feces samples under a carbohydrate analyzer. The list of analyzed polysaccharides in each group is presented in Table 3. Briefly, Ankom technology was used to analyze NDF, ADF, and ADL sequentially using an Ankom 220 Fiber Analyzer. For the determination of NDF, a heat-stable amylase was used as described by Mertens [55]. Afterward, a correction was made for ash using the ash residue obtained after ADL determination. The collected feces samples were freeze dried for 72 hr and homogenized before analysis.

The microminerals, yttrium oxide, and iodine concentrations in diets, and pooled samples of whole body, muscle, and feces were determined by inductively coupled plasma mass spectrometry (ICP-MS), as described by Long and Martin [56] and Julshamn et al. [57]. In brief, for determination of the microminerals, 0.2 g freeze-dried sample material was digested in a microwave oven (Milstone-MLS-1200), diluted to 25 mL with Milli-Q Water, and analyzed using ICP-MS (Agilent 7500c). For the determination of iodine, the sample preparation was a basic extraction with tetramethylammonium hydroxide (TMAH) before ICP-MS analysis.

### 2.7. Performance Calculations

The following variables were calculated [58]:

(1) $$\begin{array}{c} \text{Digestible energy }\left(DE,\frac{MJ}{kg}\right)=\text{Energy}\in \text{Diet}-\left(\frac{\text{Yttrium}\in \text{Diet}}{\text{Yttrium}\in \text{Faeces}}\times \text{Energy}\in \text{Faeces}\right), \end{array}$$

(2) $$\begin{array}{c} \text{Weight gain }\left(\text{WG},g\right)=\text{Final mean weight }\left(g\right)-\text{Initialmeanweight}\left(g\right), \end{array}$$

(3) $$\begin{array}{c} \text{Specific growth rate }\left(\text{SGR},\%\text{ per day}\right)=\left(\text{ln final BW}-\text{lninitial BW}\right)\times \frac{100}{t}. \end{array}$$

As described by Hopkins [59], where ln final BW and ln initial BW are the natural logarithm of final and initial biomass in grams and *t* is the sum of feeding days (70 days). In the current study, the mean SGR of the fish from mixed population was determined for each tank. In addition, individual SGR was also calculated on the 10 pit-tagged fish from the all-male population per tank.

(4) $$\begin{array}{c} \text{Feed conversion ratio }\left(\text{FCR}\right)=\frac{\text{Feed intake}}{\text{Weight gain}}. \end{array}$$

As described by Helland et al. [43], total feed intake was calculated as an estimate of DM content of the waste feed (obtained in the recovery test):

(5) $$\begin{array}{c} \text{Total feed intake }\left(\text{TFI},g\right)=\frac{\left(\frac{A\times \text{ADW}}{100}\right)-\left(\frac{W\times \text{WDW}}{R}\right)}{\frac{\text{ADW}}{100}}, \end{array}$$

where *A* is the weight of air-dry feed (g), ADW is the DM content of air-dry feed (%), *W* is the wet weight of waste feed collected (*g*), WDW is the DM content of waste feed (%), and *R* the is recovery of DM of waste feed (%) that was calculated as follows:

(6) $$\begin{array}{c} \text{Recovery }\left(R,\%\right)=100\times \frac{W\times \text{WDW}}{A\times \text{ADW}}. \end{array}$$

Average daily feed intake per kg biomass (DFI–% biomass) was calculated from recorded daily feed intake and estimated daily biomass from SGR using the following equation:

(7) $$\begin{array}{c} \text{ln }W\text{ day}x=\left(\frac{\text{SGR}}{100}\right)\times \left(1+\text{ln }W\text{ day }\left(x-1\right)\right), \end{array}$$

where ln *W* day*x* is the natural logarithm of biomass on a given day [60].

(8) $$\begin{array}{c} \text{Condition factor}\left(K,\frac{g}{cm^{3}}\right)=100\times \frac{\text{Body weight }\left(g\right)}{\text{Body length }\left(cm^{3}\right)}. \end{array}$$

The hepatosomatic indexes (HSI), cardio somatic indexes (CSI), and visceral somatic indexes (VSI) were calculated as percentages of the final weight:

(9) $$\begin{array}{c} \text{Hepatosomatic index }\left(HSI,\%\right)=100\times \left(\frac{\text{Liver weight}}{\text{Whole body weight}}\right), \end{array}$$

(10) $$\begin{array}{c} \text{Cardiosomatic index }\left(CSI,\%\right)=100\times \left(\frac{\text{Heart weight}}{\text{Whole body weight}}\right), \end{array}$$

(11) $$\begin{array}{c} \text{Viscerosomatic index }\left(VSI,\%\right)=100\times \left(\frac{\text{Viscera weight}}{\text{Whole body weight}}\right). \end{array}$$

To understand how much of the ingested feed ingredient is absorbed by the animal and retained in their body, the apparent digestibility/availability coefficient (ADC/AAC), and retention of nutrients were measured as described by Cho [61]:

(12) $$\begin{array}{c} ADC\;\left(\%\right)=100-\left(100\times \frac{\text{Yttrium }in\;diet}{\text{Yttrium }in\text{ faeces}}\times \frac{\text{Nutrient }in\text{ faeces}}{\text{Nutrient }in\;diet}\right), \end{array}$$

(13) $$\begin{array}{c} AAC\;\left(\%\right)=100-\left(100\times \frac{\text{Yttrium }in\;diet}{\text{Yttrium }in\text{ faeces}}\times \frac{\text{Mineral }in\text{ faeces}}{\text{Mineral }in\;diet}\right). \end{array}$$

Nutrient retention (%) was calculated from fish biomass and nutrient content of the fish at the start and end of each growth period and nutrient intake:

(14) $$\begin{array}{c} \text{Retention }\left(\%\right)=100\times \frac{\left(BM\;f\times \text{Nutrient content }f\right)-\left(BM\;i\times \text{Nutrient content }i\right)}{\text{Feed intake}\times \text{Nutrient in feed}}, \end{array}$$

where f and i are the nutrient content in final and initial, respectively.

### 2.8. Data Analysis

As the trial study was performed in a dose–response design, linear and nonlinear regression analyses were used to evaluate dose-dependent responses by determining the best-fit line for each dataset. In addition, one-way ANOVA was performed to assess statistically significant differences among experimental groups, and if the data were significant different, then followed up with Tukey' s multiple comparison post hoc analysis. For all datasets, Bartlett/Brown–Forsythe's test was used to assess the homogeneity of variance and Shapiro–Wilk's test was used to check the normality residuals. The ROUT test was done for the identification and removal of the outliers of the growth dataset. One of the 4% FSK tanks was removed as outlier. Tank was used as the experimental unit in growth, whole-body proximate, and mineral composition (*n* = 3 for all the experimental diets and *n* = 2 for FSK4% group). Whole body, muscle proximate composition, and mineral status of Atlantic salmon postsmolts from the all-male population were only analyzed for control and high level of FSK4%. All the statistical analyses and the graphs were performed in GraphPad Prism (Version 8.4.3 (686) San Diego, California, USA). Significance was set at *P* < 0.05 for all statistical tests, and the value is presented as mean ± SEM.

## 3. Result

### 3.1. Fish Performance Indicators

**Figure 1:**
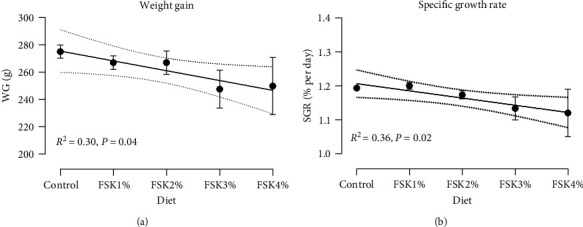
(a) Weight gain and (b) specific growth rate (SGR) of Atlantic salmon postsmolt fed graded inclusion of fermented sugar kelp (FSK). The best-fit regression lines for each dataset were presented (the regression equations are presented in Table 5). Values are presented as mean ± SEM, all diets are in triplicate except FSK4%, that is, in duplicate, *n* = 15 fish per diet.

During the experiment, the fish almost doubled the weight in all experimental groups (Table 5). Total feed intake (the mean of all experimental groups, 12.3 ± 0.2 kg) and feed conversion ratio (FCR) (0.7 ± 0.02) were not affected by FSK inclusion levels in experimental diets. However, WG and SGR decreased in a dose–response manner under a simple linear regression (*P*=0.04 and *P*=0.02, respectively) with FSK inclusion (Figures 1(a) and 1(b)). In comparison to the control group, the WG of FSK1% and 2% decreased by 3%, FSK3% and 4% decreased by 10% and 9%, respectively. All groups had a similar *K* of around 1.2 ± 0.0. Furthermore, no dose-dependent responses were seen in morphometric measurements of the somatic indices (HSI, VSI, and CSI) among the experimental groups. At the end of the 10-week trial, the mean cataract score was below 1 (0.7 ± 0.04) for all groups, and no difference was seen among the experimental groups. Moreover, there was no difference in visually assessed welfare indicators between experimental groups (only two fish had the scale loss and short operculum with score 2).

The mean weight of the all-male postsmolts (*n* = 10 per tank, *n* = 30 per diet) was a little higher than the mixed population (*n* = 55 per tank, *n* = 165 per diet) at the end of the experiment, but it was still within the same range for both groups (Supplementary Figure S1A). The SGR of the all-male fish decreased slightly from 1.30 ± 0.03 in control group to 1.26 ± 0.04 in FSK4% group, however not significantly different (*P*=0.2). This resulted in a 5%–7% lower weight gain, but the reduction was also not significant (*P*=0.15). The growth performance results for the all-male population are also reported separately as supplementary material (Supplementary Table S1A).

#### 3.1.1. Apparent Digestibility and Apparent Availability Coefficient

No difference was seen in the digestibility of macronutrients except for total fat ADC (Table 6). Total fat ADC increased significantly from 94.4 ± 0.4% in the control group to 97.1 ± 0.8% in the 4% FSK group under a simple linear regression (*P*=0.03). The protein ADC ranged between 86.9 ± 0.6% in the control group and 88 ± 0.3% in the experimental groups. Gross energy digestibility was 77.9 ± 1.2% and 80.1 ± 0.4% for the control and experimental groups, respectively. The average of the carbohydrate ADC (control and experimental groups together) was calculated to be 66.6 ± 2.0% for NDF, −8.4 ± 2.2% for ADF, 44.0 ± 7.1% for ADL, 79.9 ± 1.5% for hemicellulose, and −16.5 ± 2.2% for cellulose.

**Figure 2:**
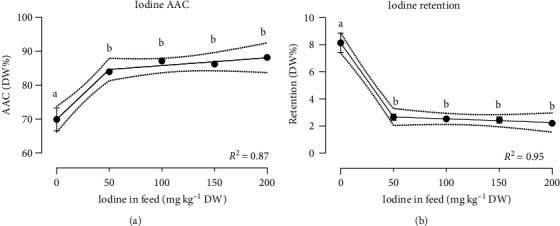
(a) Iodine apparent availability coefficient (AAC), (b) iodine retention of Atlantic salmon postsmolt fed graded inclusion of fermented sugar kelp (FSK). The best-fit regression lines for each dataset were presented (the regression equations are presented in Table 6). Statistically significant differences between the experimental groups were represented with different letters above the data points (*P* < 0.05) under the Tukey HSD test. Values are presented as mean ± SEM, all diets are in triplicate except FSK4%, that is, in duplicate.

Availability of iodine significantly increased with FSK inclusion in the diet under a segmental linear regression response with a broken point in FSK1% (Figure 2(a)). The iodine availability increased from 69.9 ± 3.4% in the control group to 86.0 ± 0.5% in the FSK groups. In addition, Se AAC was significantly increased by FSK diets. The 2% FSK-supplemented group had the highest Se AAC (63.4 ± 0.2%) fitted by a segmental linear regression with a broken point in FSK2% (Table 6). The availability of the other analyzed minerals Zn, Mn, Cu, and Fe was not affected by FSK inclusion in the diet (Table 6).

### 3.2. Whole Body and Muscle Composition

The total fat, energy, and DM content all showed a dose-dependent response (*P*=0.001, *P*=0.003, *P*=0.01, respectively) and decreased with FSK inclusion in diet (Table 7). The 3% and 4% FSK groups had the lowest amount of total fat, gross energy, and DM in body compared with the other groups. No effect was found on the protein and ash body composition by adding the FSK to the salmon diet.

**Figure 3:**
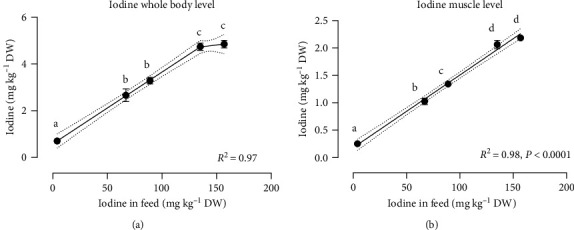
(a) Whole body and (b) muscle iodine status of Atlantic salmon postsmolt fed graded inclusion of fermented sugar kelp (FSK). The best-fit regression lines for each dataset were presented (the regression equations are presented in Table 7 in a WW). The whole body and muscle iodine concentrations are presented in DW in the graphs (a) and (b). The regression formula for graph (a) is a segmental linear regression, *Y*_1_ = 0.03071*x* + 0.5849, *Y*_2_ = 0.005678 (*x*−135) + 4.73075, *Y* = IF (*X* < 135, *Y*_1_, *Y*_2_), *X*_0_ = 135 and for graph (b) is a simple linear regression, *Y* = 0.01326*x* + 0.1810. Statistically significant differences between the experimental groups were represented with different letters above the data points (*P* < 0.05) under the Tukey HSD test. Values are presented as mean ± SEM, all diets are in triplicate except FSK4%, that is, in duplicate.

The concentration of iodine in the fish whole body increased in a dose-dependent manner with increasing iodine level in the feed Figure 3(a). The iodine level was 0.2 ± 0.0 mg kg^−1^ WW in the fish fed the control feed, while it increased around 7.5 times, and reached 1.5 ± 0.1 mg kg ^−1^ WW in the whole body of fish fed FSK3%, where the levels appeared to plateau. Whole-body Cu concentration decreased from 1.6 ± 0.0 to 1.3 ± 0.1 mg kg^−1^ WW under a simple linear regression response (*P*=0.03).

Muscle iodine level increased almost six-fold from 0.1 ± 0.0 mg kg^−1^ WW in the control group to 0.6 ± 0.0 mg kg^−1^ WW in the FSK4% group under a simple linear regression response (*P* < 0.0001, Figure 3(b)). No significant differences were observed in other essential micromineral (Mn, Fe, Se, and Zn) concentrations in whole body and muscle among dietary treatments.

The nutritional status of the fish from the all-male population was determined in both the control and FSK4% groups and is included in Supplementary Table S1B. A similar pattern of nutritional status was seen between both mixed and all-male populations.

### 3.3. Retention of Nutrient and Essential Elements

The retention of total fat, gross energy, and DM decreased with a higher FSK inclusion (Table 8). The highest fat retention was seen in the 4% FSK-supplemented group (82.0 ± 8.0%) under a second polynomial model. However, the energy and DM retention decreased with a higher FSK inclusion under a simple linear regression (*P*=0.04 and *P*=0.03, respectively). There was no effect of FSK diets on protein and ash retention.

Among the microminerals, Cu and iodine retention decreased with increasing inclusion of FSK into the diet and presented a dose-dependent response (Table 8, Figure 2(b)). Copper retention was reduced from 23.1 ± 0.9% in control group to 12.8 ± 0.0% in 4% FSK-supplemented group under a simple linear regression response (*P*=0.01). Furthermore, iodine retention decreased significantly by adding 1% of FSK to the diet and followed almost a plateau pattern fitted by a segmental linear regression with a broken point in FSK1% (Figure 2(b)). The retention of the other minerals was not affected by the inclusion of FSK into the diet and dose-dependent responses were not observed (Table 8).

## 4. Discussion

The present study was conducted to determine the effect of adding increasing levels of FSK in the diet of farmed Atlantic salmon. Since the diets were made to simulate a standard grower feed for salmon postsmolts in SW, the feeds were made with ∼63% plant-based ingredients vs.∼34% marine ingredients. Although sugar kelp is not a considerable source of lipids and proteins for Atlantic salmon, the potential in using this low-trophic marine biomass in aquafeeds is interesting mostly as a source of bioactive compounds, but possibly also as a source of minerals [9, 10]. The high level of indigestible carbohydrates does, however, raise concerns about using it in the feed for salmon, along with the contribution of high levels of iodine. Thus, the present study aimed to investigate how the use of FSK in the diet for Atlantic salmon may modulate growth, welfare, digestibility of nutrients, and retention of nutrients with special emphasis on iodine.

Historically, the use of novel feed ingredients has sometimes resulted in the occurrence of production-related disorders and welfare issues, that could for instance be related to single nutrient deficiencies or toxicities, as well as lower bioavailability of nutrients in novel resources [62]. In the present study, the fish were fed experimental diets for 10 weeks, and in this period, they more than doubled their weight from around 200 g to the range of 500 g. The inclusion of FSK slightly reduced the SGR from 1.2% to 1.1%, resulting in approximately 3% lower weight gain in the FSK1% and 2% groups, and a weight reduction of 10% and 9% in the FSK3% and 4% groups, respectively, compared to the control group Figures 1(a) and 1(b). A similar response was seen in rainbow trout fed a diet containing 4% sugar kelp, however in that study, including 1% and 2% sugar kelp in the diet did not reduce growth performance [28]. Despite the reduction in weight gain, no differences were observed in daily feed intake and FCR between the experimental groups in the present study, showing that feed utilization was not reduced by including FSK in the diet. The latter is of high importance considering the climate footprint of feed ingredients that is also dependent on the ability of the fish to utilize the feed [29, 30]. Further, the occurrence of production disorders and welfare issues resulting from nutritional deficiencies and possible related pathologies and losses also contribute to the sustainability evaluation of a raw material. At the end of the feeding study, a visual inspection of outer welfare indicators [45] as well as assessment of eye lens health (cataract score) could not identify any nutritionally related pathologies in any of the experimental groups. Therefore, while the inclusion of FSK in the diet resulted in a slight reduction in fish growth, there was no impact on feed utilization and fish welfare.

Previous studies have indicated that using high levels of macroalgae in fish feed influences the digestibility of nutrients, which in turn can be a potential cause of growth impairment [22]. This effect is attributed to the presence of high levels of complex polysaccharides, that can increase the passage of food through the digestive tract and consequently also reduce nutrient absorption [25, 63, 64]. However, in the present study, the dietary carbohydrate composition was similar across all experimental diets. The diets containing FSK had lower concentrations of NDF and hemicellulose, ranging from 13 to 15 g 100 g^−1^ compared to 17 g 100 g^−1^ in the control diet. Due to the contribution of fiber from the plant-based ingredients used in the feed, the inclusion of FSK led to a decrease in the overall carbohydrate content of the feed. Earlier studies have also shown that including various types and different inclusion levels of macroalgae could result in reduced protein digestibility. For instance, this was shown in two previous studies using dry algae meal from different macroalgae (5% Verdemin, 5% Rosamin) in the diet for Atlantic salmon [8] and sugar kelp at similar levels as in the present study (1%, 2%, and 4%) in the diet for rainbow trout [28]. One of the suggested reasons for the decreased protein ADC was the poor ability of fish to digest algae-derived proteins in addition to a limited ability to hydrolyze complex polysaccharides [65], which caused a maximum protein ADC of 45%–56% in fish [66]. It has been shown that including indigestible carbohydrates such as NSP in fish diet, for example tilapia diet, decreased the digestibility of proteins and lipids by impairing the fish's ability to absorb minerals and water and raising the viscosity of the digesta [67, 68]. In contrast to these findings, indigestible carbohydrates did not cause any problem in the current study, and it was found that adding FSK enhanced the fat digestibility (by 3%) while leaving the protein and carbohydrate digestibility unaffected. Since there was no significant increase in dietary carbohydrate or difference in feed consumption, protein, and carbohydrate ADC with increasing levels of FSK, it is unlikely that the observed reduction in growth can be attributed to these factors.

The lower dietary lipid level in the FSK4% diet compared with the other experimental diets (28% lower than the control group), likely resulted in a higher lipid digestibility and a relatively higher retention of total fat in the FSK4% group, while the whole-body fat content in this group was 14% lower than the control group. These changes were not reflected in the somatic indexes, which were contrary to the study by Granby et al. [28] which showed a negative correlation between sugar kelp inclusion in the rainbow trout diet and HSI, the HSI of rainbow trout fed 4% sugar kelp significantly decreased. The excess energy is stored in the liver, and HSI is used as an indirect indicator for measuring the energy status. The inclusion of FSK did, however, result in a lower dietary energy level and DE, and the reduction in growth and weight gain may rather be due to the overall energy dilution caused by incorporating FSK in the diets, which was also reflected in the whole-body composition of fat, energy, and DM. As a challenge of using macroalgae in monogastric animal feed, it has been observed that the high content of polysaccharide components such as alginate and carrageenan resulted in lower nutritionally available energy content of macroalgae and most algae-derived products [2, 69].

Sugar kelp contains a high level of iodine as reported by previous studies (up to 4,600 mg kg^−1^ DW) [32, 70, 71]. This high iodine content is a concern when considering kelp inclusion in fish diets. Notably, the upper tolerance level and no-observable-adverse-effect level (NOAEL) for dietary iodine have not been determined in farmed fish, while it has been proposed that the tolerances are 3–10-fold higher than the requirement [72]. In commercial aquaculture feed iodine is generally derived from fish meal and added as potassium iodide in the mineral premix [73]. Due to the generally lower iodine content of plant-based feeding stuff, incorporating them into fish diets may require an increased need for iodine supplementation [72]. The requirement of salmonids for dietary iodine is relatively low (1.1 mg kg^−1^) [40, 74], and the maximum recommended level for iodine salt in farmed fish diets is 20 mg iodine kg^−1^ (based on 880 g kg^−1^ DW) [75] which still result in lower tissue concentrations in farmed fish compared with wild marine fish [72]. However, it was shown the maximum tolerable dietary iodine level is higher than 60 mg iodine kg^−1^ in farmed fish [72, 76]. Feed containing up to 86 mg iodine (as potassium iodine, KI) kg^−1^ diet fed to adult Atlantic salmon (*Salmo salar* L.) [77], and 2% kelp diet with an concentration of 117 ± 2 mg iodine kg^−1^ diet fed to rainbow trout (*Oncorhynchus mykiss*) [28] had no adverse effects on growth performance, and health of these species. In the current study, the addition of sugar kelp increased the dietary iodine content from 4 mg kg^−1^ WW in the control feed up to 138 mg kg^−1^ WW in the 4% FSK feed. Due to the overall low dietary energy that influenced growth performance, it is challenging to determine if the high dietary iodine level caused the growth reduction. Nevertheless, the increase in iodine AAC and improvement in iodine body status until reaching a plateau level (FSK3% containing 124 mg kg^−1^ WW) suggested a possible regulation of iodine uptake and deposition in the fish body. Additionally, exposure to high dietary iodine levels resulted in a decrease in iodine retention. These results indicated that fish have a mechanism to adjust their iodine metabolism in response to high dietary iodine levels. This finding is consistent with previous research which has shown that certain species of fish are capable of efficiently excreting excess metals and maintaining normal levels of concentration in their bodies [78].

Previous studies have shown that the dietary iodine concentrations can be reflected in the muscle iodine level [28, 79–81]. In line with that, in the present study, the muscle iodine level for Atlantic salmon fed FSK1% and 2% (60 and 80 mg iodine kg^−1^ WW, respectively) was around 0.3 ± 0.0 mg kg^−1^ WW (four-fold of control diet) and reached around 0.6 ± 0.0 mg kg^−1^ WW in the muscle of fish fed FSK3% and 4% (124 and 138 mg iodine kg^−1^ WW). However, in the study by Granby et al. [28], the muscle iodine level in rainbow trout exhibited a four-fold increase, rising from 0.3 ± 0.08 mg kg^−1^ WW in fish fed 1% sugar kelp (57 mg iodine kg^−1^ WW) to 1.2 ± 0.45 mg kg^−1^ WW in fish fed 4% sugar kelp (220 mg iodine kg^−1^ WW). It is important to note that Granby et al. [28] included the skin in their muscle samples, whereas the current study did not, which could account for the conflicting results between the two studies. It has been shown that the skin of freshwater Char (*Salvelinus* sp.) displayed a five-fold higher iodine concentration compared to the skinless muscles [79]. Additionally, a higher dietary iodine level in the diet containing 4% sugar kelp was utilized in the study by Granby et al. [28], further contributing to the differences.

The sugar kelp used in this study had a Se concentration of less than 0.008 mg kg^−1^ WW (below detection limit), and just above the detection limit (0.01 mg kg^−1^ WW) after the fermentation, which was consistent with the findings of Bruhn et al. [38]. Although the dietary Se levels met the minimum Se requirement (0.6–0.8 mg kg^−1^ DW) [82], a high level of FSK in the diet led to an 11% and 22% decrease in dietary Se level for FSK3 and 4%, respectively, when compared to the control diet. Brown seaweeds typically have low levels of selenium [70], and it is possible that when included in fish feed, this may dilute the selenium content of the overall diet. The apparent availability of Se was higher in the diet containing 2% FSK; however, this did not translate into increased Se retention or whole-body status. These findings are contrary to the study by Granby et al. [28], which showed decreased Se AAC with the incorporation of sugar kelp (1%, 2%, and 4%) in rainbow trout diets. However, the differences in the results may be related to the overall impact on digestibility and nutrient retention in the study with rainbow trout that was not seen in the present study.

The inclusion of FSK negatively affected the distribution and retention of Cu in the whole body. This may be attributed to the higher level of iodine in the fish, as both iodine deficiency and oversupply can disrupt mineral (e.g., Cu, Mn, Fe, and Zn) homeostasis [83, 84].

## 5. Conclusion

Overall, the incorporation of FSK in the experimental diets reduced the growth, which may be related to the overall lower energy content in these feeds since feed intake and feed utilization (FCR) were similar. The use of FSK did not influence the digestibility of macronutrients except for lipids. The retention of lipid, energy, and DM was reduced with FSK inclusion in diet, which corresponded with whole-body macronutrient composition. Apparent mineral availability (except iodine and Se) and mineral retention (except iodine and Cu) were not affected by FSK inclusion by up to 4%. The incorporation of FSK in the diets improved iodine availability. Our results indicated that up to 3% FSK supplementation in the Atlantic salmon diet has the potential to improve the muscle iodine concentration. Up to 2% FSK inclusion in the postsmolt salmon diet improved Se availability. FSK inclusion in the diet of Atlantic salmon had no influence on the welfare indices studied.

## Figures and Tables

**Table 1:** Several macroalgae species used as feed supplement or replacement.

Fish species	Macroalgae species	Tested levels	Best level	Substituted ingredient	Effect	Reference
European sea bass (*Dicentrarchus labrax*)	*Gracilaria bursa-pastoris*	5%–10%	10%	Fish protein hydrolysate	No negative consequences on growth performance, nutrient utilization, or body composition 10% inclusion of *Gracilaria cornea* significantly decreased growth performance, dry matter, and lipid ADC and increased FCR	[85]
	*Gracilaria cornea*		5%			
	*Ulva rigida*		10%			

Gilthead sea bream (*Sparus aurata*)	*Pterocla dia*	5%–15%	10%	Wheat flour	Up to 10% *Pterocladia* and 5% *Ulva* showed the best growth performance, feed utilization, nutrient retention, and survival 15% inclusion reduced the growth and feed utilization	[86]
	*Ulva*		5%			

Red sea bream (*Pagrus major*)	*Ascophyllum nodosum*	5%	5%	Protein	Increased body weight, feed efficiency, and muscle protein deposition Feeding *Porphyra* showed the most pronounced effects on growth and energy accumulation, followed by *Ascophyllum* and *Ulva*	[87]
	*Porphyra yezoensis*					
	*Ulva pertusa*					

Red sea bream (*Pagrus major*)	*Wakame or Ascophyllum*	5%–10%	5%	Protein, rice, and/or wheat bran	The best growth and feed efficiency and higher muscle lipid content in the 5% Wakame diet group	[88]

Rainbow trout *(Oncorhynchus mykiss)*	*Porphyra dioica*	5%–15%	Up to 10%	Fish meal and wheat starch	No significant negative effects on weight gain and growth performance	[89]

Rainbow trout (*Oncorhynchus mykiss*)	*Gracilaria vermiculophylla*	5%–10%	Up to 5%	Fish meal	No effect on growth or FCR, increased iodine and moisture content, and higher color intensity 10% inclusion resulted in significantly smaller fish	[80]

Rainbow trout (*Oncorhynchus mykiss*) and Nile tilapia (*Oreochromis niloticus*)	*Porphyra dioica*, *Ulva spp*. *Gracilaria vermiculophylla* *Sargassum muticum*	30%	30%	Mixing 70% basal diet and 30% of each seaweed	Trout digested better Gracilaria, while Nile tilapia does better with Ulva and Sargassum	[24]

Rainbow trout (*Oncorhynchus mykiss*)	*Ulva lactuca* *Enteromorpha linza*	10%	—	Added to the diet	Poor growth and feed utilization	[90]

Rainbow trout (*Oncorhynchus mykiss*)	*Gracilaria vermiculophylla*	5%–10%	Up to 5%	Several dietary protein source	No difference in growth, FCR and protein efficiency ratio, and increased innate immune response 10% inclusion resulted in the lowest final body weight, feed and protein efficiency, and protein retention	[26]

Rainbow trout (*Oncorhynchus mykiss*)	Sugar kelp *(Saccharina latissima)*	1%– 4%	Up to 2%	Wheat meal	No effect on growth performance, increased protein efficiency, and increased iodine level in fillet 4% inclusion resulted in lower growth, lower hepatosomatic indices, and histomorphological change in intestine	[28]

Atlantic salmon (*Salmo salar*)	Laminaria sp., kelp	3%–10%	Up to 10%	Fish meal	Increased food consumption, enhance growth performance, improve antioxidant capacity, and alleviate adverse effects of stressors such as temperaturte	[91]

Atlantic salmon (*Salmo salar*)	Verdemin	2.5%–5%	Up to 5%	Added to the diet	No effect on growth and feed efficiency	[8]
	Rosamin	2.5%–5%	5%		Increased omega3 long-chain polyunsaturated fatty acid in whole body	
	Mixing Verdemin and Rosamin	2.5% of each	Up to 5%		No effect on growth and feed efficiency	
Atlantic salmon (*Salmo salar*)	*Palmaria palmata*	5% –15%	Up to 15%	Starch and fish meal	No effect on fish growth, no effect on haematological, immunological, and hepatic function. Five percent inclusion improved body lipid content Alanine transaminase activity significantly decreased in the diet with 5% and 15% *Palmaria palmata*	[92]

**Table 2:** Macronutrient and mineral proximate composition of fresh and fermented sugar kelp (FSK).

	Fresh sugar kelp	Fermented sugar kelp (after 3 weeks)
Macronutrients proximate composition (g 100 g^−1^ WW)		
Crude protein	1.3	1.3
Fat	<1	<1
Ash	4.1	3.8
Dry matter	8.7	8.6
Carbohydrate composition (% WW)		
T-NCP^1^	1.0	0.8
T-NSP^2^	1.4	1.2
Cellulose	0.4	0.4
Lignin-like substance	0.2	0.1
S-DF^3^	0.8	0.7
I-DF^4^	0.7	0.6
T-DF^5^	1.5	1.3
Micromineral composition (mg kg^−1^ WW)		
Mn	0.5	0.5
Fe	7.0	5.0
Cu	0.2	1.2
Zn	3.0	3.0
Se	<0.008	0.01
I	430	400

Data are given as mean ± SEM (*n* = 3). WW refers to a wet weight basis. ^1^T-NCP stands for total noncellulosic polysaccharide that contains soluble and insoluble noncellulosic polysaccharides. Soluble and insoluble noncellulosic polysaccharides are rhamnose, fucose, arabinose, xylose, mannose, galactose, glucose, and uronic acid. ^2^T-NSP stands for total nonstarch polysaccharides that contain soluble and insoluble nonstarch polysaccharides. Soluble nonstarch polysaccharides are equal to soluble noncellulosic polysaccharides and insoluble nonstarch polysaccharides contains insoluble noncellulosic polysaccharides and cellulose. ^3^S-DF stands for soluble dietary fiber that contains soluble noncellulosic polysaccharides. ^4^I-DF stands for insoluble dietary fiber that contains insoluble noncellulosic polysaccharide, cellulose, and lignin. ^5^T-DF stands for total dietary fiber that contains total nonstarch polysaccharide and lignin.

**Table 3:** Formulation (in % of total raw materials) and proximate composition of the experimental diets containing different levels of fermented sugar kelp (FSK).

	Control	FSK1%	FSK2%	FSK3%	FSK4%
Fish oil	10.2	10.3	10.4	10.5	10.6
Rapeseed oil	13.9	13.6	13.4	13.2	12.9
Fishmeal LT	25.0	23.3	21.6	19.9	18.2
Soy protein concentrate (SPC)	20	20	20	20	20
Wheat	11.0	11.0	10.9	10.5	10.0
Other plant proteins^1^	16.8	17.5	18.3	19.4	20.6
Microingredients	3.2	3.3	3.4	3.5	3.6
Yttrium oxide	0.02	0.02	0.02	0.02	0.02
Fermented seaweed	-	1	2	3	4
Analyzed proximate composition (g 100 g^−1^ WW)					
Protein	46	45	43	44	46
Lipid	25	25	24	22	18
Ash	7	7	7	7	8
Gross energy (MJ kg^−1^ WW)	23	22	23	22	21
Digestible energy (MJ kg^−1^ WW)	19	18	19	18	18
Dry matter	95	93	94	95	92
Carbohydrate (g 100 g^−1^ WW)					
NDF^2^	16	14	13	13	13
ADF^3^	2	1.9	1.9	2.1	2.1
ADL^4^	0.3	0.3	0.2	0.3	0.3
Hemicellulose	14	12	11	11	11
Cellulose	1.7	1.7	1.7	1.8	1.8
Mineral composition (mg kg^−1^ WW)					
Mn	51	48	51	52	52
Fe	190	186	197	181	193
Cu	10	9	9	10	10
Zn	162	149	150	162	156
Se	0.8	0.8	0.8	0.8	0.7
I (DW)	4 (4)	60 (67)	80 (89)	124 (135)	138 (157)

*Notes*: Ingredients are listed as percentages of whole feed. WW and DW refer to wet weight and dry weight basis. ^1^Wheat gluten meal, pea protein concentrate- and guar-meal. ^2^NDF stands for neutral detergent fiber and contains soluble NDF (sugars, pectin, nonprotein *N*, soluble protein) and insoluble NDF (hemicellulose, fiber-bound protein, cellulose, lignin, lignified *N*). ^3^ADF stands for acid detergent fiber and contains soluble ADF (hemicellulose, fiber-bound protein) and insoluble ADF (cellulose, lignin, and lignified *N*). ^4^ADL stands for acid detergent lignin and contains soluble ADL (cellulose) and insoluble ADL (lignin, cutin).

**Table 4:** Amino acids composition of the experimental diets containing different levels of fermented sugar kelp (FSK).

(mg g^−1^ as is)	Control	FSK1%	FSK2%	FSK3%	FSK4%
Hydroxyproline	1.9	1.6	1.6	1.5	1.5
Histidine	12.5	11.8	12.0	12.0	12.6
Taurine	1.5	1.3	1.3	1.2	1.2
Serine	19.7	18.9	18.7	19.4	20.5
Arginine	28.3	26.8	26.8	27.2	29
Glycine	21.2	19.6	19.6	19.6	20.5
Aspartic acid	40.0	39.0	38.0	38.0	41.0
Glutamic acid	74.0	73.0	73.0	75.0	82.0
Threonine	15.8	15.0	14.8	15.0	15.8
Alanine	19.7	18.7	18.4	18.4	19.5
Proline	21.7	21	21.1	21.8	23.6
Lysine	26.8	25.4	24.8	24.5	26.2
Tyrosine	13.9	13.2	13.2	13.8	14.2
Methionine	12.0	11.3	11.1	11.4	11.9
Valine	18.8	17.8	17.9	17.7	19.1
Isoleucine	17.1	16.2	16.4	16.1	17.5
Leucine	31.0	29.1	29.0	29.2	31.0
Phenylalanine	19.9	18.9	19.3	19.6	20.7

*Notes*: WW refers to wet weight basis.

**Table 5:** Growth performance indicators of Atlantic salmon postsmolts and fed graded inclusion levels of fermented sugar kelp (FSK).

	Control	FSK1%	FSK2%	FSK3%	FSK4%	Regression (*P* value, *R*^2^)	ANOVA
IBW (g)	210.1 ± 3.5	203.1 ± 1.1	208.8 ± 3.4	203.9 ± 2.3	209.1 ± 0.5	n.s.	n.s.
FBW (g)	485.1 ± 8.0	470.1 ± 6.1	475.8 ± 12.0	451.5 ± 16.1	458.9 ± 20.5	n.s.	n.s.
WG (g)	275.0 ± 4.8	267.0 ± 5.0	267.0 ± 8.5	247.6 ± 13.9	249.8 ± 21.0	*P*=0.04, *R*^2^ = 0.30^1^	n.s.
SGR (% per day)	1.2 ± 0.0	1.2 ± 0.0	1.2 ± 0.0	1.1 ± 0.0	1.1 ± 0.1	*P*=0.02, *R*^2^ = 0.36^2^	n.s.
TFI (kg)	11.9 ± 0.2	12.6 ± 0.5	12.8 ± 0.1	12.3 ± 0.7	11.9 ± 0.1	n.s.	n.s.
DFI (% of biomass)	0.8 ± 0.0	0.8 ± 0.1	0.8 ± 0.0	0.8 ± 0.1	0.8 ± 0.0	n.s.	n.s.
FCR	0.7 ± 0.0	0.7 ± 0.0	0.7 ± 0.0	0.8 ± 0.1	0.7 ± 0.1	n.s.	n.s.
K	1.3 ± 0.0	1.2 ± 0.0	1.2 ± 0.0	1.2 ± 0.0	1.2 ± 0.0	n.s.	n.s.
HSI	1.1 ± 0.1	1.0 ± 0.0	1.0 ± 0.0	1.1 ± 0.0	1.0 ± 0.0	n.s.	n.s.
CSI	0.2 ± 0.0	0.2 ± 0.0	0.2 ± 0.0	0.2 ± 0.0	0.2 ± 0.0	n.s.	n.s.
VSI	7.2 ± 0.4	6.9 ± 0.1	7.0 ± 0.1	7.1 ± 0.1	7.1 ± 0.4	n.s.	n.s.
Cataract score	0.5 ± 0.1	0.6 ± 0.1	0.7 ± 0.1	0.7 ± 0.1	0.6 ± 0.1	n.s.	n.s.

*Notes*: IBW = initial body weight (g), FBW = final body weight (g), WG = weight gain (g), SGR = specific growth rate, K = condition factor, TFI = total feed intake (g), DFI (%) = daily feed intake as percentage of biomass, FCR = feed conversion ratio, HSI = hepatosomatic index, CSI = cardio somatic index, and VSI = visceral somatic index. Data are presented as mean ± SEM. The somatic indices are a mean of 15 fish per diet (five fish per tank), the weight and length data are a mean of all fish (*n* = 55 fish per tank), and the cataract is a mean of 30 fish per diet (*n* = 10 fish per tank). All diets are in triplicate except FSK4%, that is, in duplicate. n.s. stands for not significant. ^1^Simple linear regression, *Y* = − 7.190*x* + 275.4. ^2^Simple linear regression, *Y* = − 0.02122*x* + 1.207.

**Table 6:** Apparent digestibility coefficients (ADC) of macronutrients and apparent availability coefficient (AAC) of minerals of Atlantic salmon postsmolt fed graded inclusion of fermented sugar kelp.

	Control	FSK1%	FSK2%	FSK3%	FSK4%	Regression (*P* value, *R*^2^)	ANOVA
Macronutrients ADC (%)							
Crude protein	86.9 ± 0.6	88.0 ± 0.3	89.1 ± 0.3	87.3 ± 0.4	88.5 ± 0.3	n.s.	n.s.
Total fat	94.4 ± 0.4	96.3 ± 0.5	96.5 ± 0.8	96.6 ± 1.1	97.1 ± 0.8	*P*=0.03, *R*^2^ = 0.32^4^	n.s.
Digestible energy	77.9 ± 1.2	79.8 ± 0.2	81.5 ± 0.8	79.2 ± 1.1	79.7 ± 0.2	n.s.	n.s.
Structural carbohydrate ADC (%)							
NDF^1^	70.0 ± 4.6	69.5 ± 4.4	68.4 ± 2.6	62.1 ± 4.7	61.8 ± 9.3	n.s.	n.s.
ADF^2^	−13.5 ± 8.5	−7.5 ± 5.3	−6.8 ± 3.2	−7.2 ± 3.1	−6.7 ± 5.9	n.s.	n.s.
ADL^3^	58.7 ± 1.7	23.4 ± 29.7	33.6 ± 5.3	52.7 ± 8.6	55.5 ± 16.2	n.s.	n.s.
Hemicellulose	82.0 ± 4.1	82.3 ± 2.7	81.3 ± 1.5	76.4 ± 3.8	76.9 ± 6.8	n.s.	n.s.
Cellulose	−24.9 ± 8.5	12.5 ± 2.0	−12.6 ± 4.4	−16.9 ± 2.7	−15.4 ± 3.6	n.s.	n.s.
Micromineral AAC (%)							
Zn	23.9 ± 3.4	26.9 ± 1.5	27.8 ± 1.9	23.1 ± 5.8	26.5 ± 2.7	n.s.	n.s.
Mn	−29.1 ± 18.5	−4.3 ± 3.3	−3.6 ± 6.6	−10.6 ± 13.8	−1.9 ± 7.0	n.s.	n.s.
Cu	42.2 ± 1.0	43.9 ± 2.2	45.3 ± 0.8	35.8 ± 2.4	40.3 ± 0.6	n.s.	n.s.
Fe	−3.7 ± 5.9	9.1 ± 1.5	15.3 ± 1.9	−5.5 ± 8.3	−3.3 ± 1.2	n.s.	n.s.
Se	54.9 ± 1.5^a^	57.6 ± 1.7^ab^	63.4 ± 0.2^b^	57.2 ± 2.1^ab^	54.9 ± 1.2^a^	*R* ^2^ = 0.64^5^	*P*=0.01
Iodine	69.9 ± 3.4^a^	84.0 ± 0.7^b^	87.2 ± 0.8^b^	86.2 ± 0.7^b^	88.2 ± 0.0^b^	*R* ^2^ = 0.87^6^	*P*=0.0008

*Notes*: Data are listed as mean ± SEM. The mean is from *n* = 3 pooled feces sample per diet (*n* = 65 fish per tank). All diets are in triplicate except FSK4%, that is, in duplicate. n.s. stands for not significant. ^1^NDF stands for neutral detergent fiber and contains soluble NDF (sugars, pectin, nonprotein *N*, and soluble protein) and insoluble NDF (hemicellulose, fiber-bound protein, cellulose, lignin, lignified *N*). ^2^ADF stands for acid detergent fiber and contains soluble ADF (hemicellulose, fiber-bound protein) and insoluble ADF (cellulose, lignin, lignified *N*). ^3^ADL stands for acid detergent lignin and contains soluble ADL (cellulose) and insoluble ADL (lignin, cutin). ^4^Simple linear regression, *Y* = 0.5856*X* + 95.04. ^5^Segmental linear regression, *Y*_1_ = 3.99*x* + 54.44, *Y*_2_ = 4.148 (*x*−2) + 62.42, *Y* = IF (*X* < 2, *Y*_1_, *Y*_2_), *X* _0_ = 2 (FSK2%). ^6^Segmental linear regression, *Y*_1_ = 14.73*x* + 69.92, *Y*_2_ = 1.152 (*x*− 1) + 84.65, *Y* = IF (*X* < 67, *Y*_1_, *Y*_2_), *X*_0_ = 67 (iodine in FSK1% diet).

**Table 7:** Whole body and muscle proximate composition and mineral status of Atlantic salmon postsmolt fed graded inclusion of fermented sugar kelp.

	Control		FSK1%	FSK2%	FSK3%	FSK4%	Regression (*P* value, *R*^2^)	ANOVA
Macronutrient in whole body (g 100 g^−1^ WW)								
Protein	18.3 ± 0.3		18.0 ± 0.0	17.7 ± 0.3	18.0 ± 0.0	18.0 ± 0.0	n.s.	n.s.
Total fat	13.5 ± 0.4		13.0 ± 0.2	12.9 ± 0.3	12.2 ± 0.3	12.1 ± 0.2	*P*=0.001, *R*^2^ = 0.58^1^	n.s.
Energy (J g^−1^ WW)		9407.0 ± 104.0	9357.0 ± 150.6	9180.0 ± 83.9	8917.0 ± 138.7	8975.0 ± 35.0	*P*=0.003, *R*^2^ = 0.52^2^	n.s.
Ash	1.6 ± 0.1		1.6 ± 0.1	1.6 ± 0.1	1.6 ± 0.0	1.7 ± 0.0	n.s.	n.s.
Dry matter	33.0 ± 0.4		32.8 ± 0.4	32.4 ± 0.3	31.6 ± 0.4	31.9 ± 0.1	*P*=0.01, *R*^2^ = 0.43^3^	n.s.
Micromineral in whole body (mg kg^−1^ WW)								
Mn	1.0 ± 0.2		0.8 ± 0.2	0.9 ± 0.1	0.8 ± 0.1	0.9 ± 0.1	n.s.	n.s.
Cu	1.6 ± 0.0		1.4 ± 0.1	1.5 ± 0.2	1.3 ± 0.0	1.3 ± 0.1	*P*=0.03, *R*^2^ = 0.3^4^	n.s.
Fe	8.4 ± 0.6		9.1 ± 0.4	8.5 ± 0.2	8.5 ± 0.1	8.6 ± 0.2	n.s.	n.s.
Se	0.2 ± 0.0		0.2 ± 0.0	0.2 ± 0.0	0.2 ± 0.0	0.2 ± 0.0	n.s.	n.s.
Zn	26.0 ± 0.6		25.7 ± 0.3	25.3 ± 0.9	26.3 ± 0.9	25.5 ± 0.5	n.s.	n.s.
Iodine	0.2 ± 0.0^a^		0.9 ± 0.1^b^	1.1 ± 0.0^b^	1.5 ± 0.1^c^	1.6 ± 0.1^c^	*P* < 0.0001, *R*^2^ = 0.97^5^	*P* < 0.0001
Micromineral in muscle (mg kg^−1^ WW)								
Mn	0.1 ± 0.0		0.2 ± 0.0	0.2 ± 0.0	0.1 ± 0.0	0.2 ± 0.0	n.s.	n.s.
Cu	0.3 ± 0.0		0.3 ± 0.0	0.3 ± 0.0	0.3 ± 0.0	0.3 ± 0.0	n.s.	n.s.
Fe	2.1 ± 0.1		2.1 ± 0.0	2.2 ± 0.0	2.0 ± 0.0	2.2 ± 0.1	n.s.	n.s.
Se	0.2 ± 0.0		0.2 ± 0.0	0.2 ± 0.0	0.2 ± 0.0	0.2 ± 0.0	n.s.	n.s.
Zn	4.7 ± 0.2		5.1 ± 0.3	5.2 ± 0.3	4.7 ± 0.3	5.8 ± 0.8	n.s.	n.s.
Iodine	0.1 ± 0.0^a^		0.3 ± 0.0^b^	0.4 ± 0.0^c^	0.6 ± 0.0^d^	0.6 ± 0.0^d^	*P* < 0.0001, *R*^2^ = 0.98^6^	*P* < 0.0001

*Notes*: Data are listed as mean ± SEM. The mean is from *n* = 3 pooled whole-body sample per diet (*n* = 5 fish per tank). All diets are in triplicate except FSK4%, that is, in duplicate. n.s. stands for not significant. WW refers to a wet weight basis. ^1^Simple linear regression, *Y* = −0.3711*x* + 13.47. ^2^Simple linear regression, *Y* = − 136.1*x* + 9433. ^3^Simple linear regression, *Y* = − 0.3611*x* + 33.06. ^4^Simple linear regression, *Y* = −0.08072*x* + 1.568. ^5^Simple linear regression, *Y* = 0.01010*x* + 0.2289. ^6^Simple linear regression, *Y* = 0.004266*x* + 0.05323.

**Table 8:** Macronutrients and mineral retention of Atlantic salmon postsmolt fed graded inclusion of fermented sugarkelp.

	Control	FSK1%	FSK2%	FSK3%	FSK4%	Regression (*P* value, *R*^2^)	ANOVA
Macronutrients (%)							
Crude protein	54.1 ± 2.1	47.0 ± 2.2	47.8 ± 1.8	48.8 ± 3.2	45.1 ± 3.2	n.s.	n.s.
Total fat	79.0 ± 4.5	67.3 ± 2.3	69.3 ± 2.8	67.3 ± 4.3	82.0 ± 8.0	*R* ^2^ = 0.45^1^	n.s.
Gross energy	57.6 ± 1.2	53.2 ± 1.9	50.9 ± 1.7	49.2 ± 3.1	51.8 ± 4.1	*P*=0.04, *R*^2^ = 0.30^2^	n.s.
Dry matter	52.0 ± 1.1	48.7 ± 2.2	47.1 ± 1.6	43.4 ± 2.8	47.0 ± 3.6	*P*=0.03, *R*^2^ = 0.32^3^	n.s.
Ash	27.6 ± 2.4	24.7 ± 3.9	25.2 ± 2.3	21.3 ± 1.4	23.6 ± 2.6	n.s.	n.s.
Micromineral (%)							
Zn	20.7 ± 0.7	19.0 ± 1.5	19.0 ± 1.7	19.0 ± 1.7	17.5 ± 0.5	n.s.	n.s.
Mn	2.8 ± 0.7	2.1 ± 0.8	2.0 ± 0.2	1.5 ± 0.4	2.1 ± 0.1	n.s.	n.s.
Cu	23.1 ± 0.9	16.7 ± 1.5	20.1 ± 4.4	14.5 ± 1.3	12.8 ± 0.0	*P*=0.01, *R*^2^ = 0.40^4^	n.s.
Fe	6.2 ± 0.7	6.4 ± 0.6	5.4 ± 0.4	6 ± 0.5	5.5 ± 0.2	n.s.	n.s.
Se	32.3 ± 2.3	32.7 ± 2.8	24.5 ± 0.7	31.4 ± 2.9	31.1 ± 1.0	n.s.	n.s.
Iodine	8.1 ± 0.7^a^	2.7 ± 0.2^b^	2.5 ± 0.1^b^	2.4 ± 0.2^b^	2.2 ± 0.0^b^	*R* ^2^ = 0.95^5^	*P* < 0.0001

*Notes*: Data are listed as mean ± SEM. The mean is from *n* = 3 pooled whole-body sample per diet (*n* = 5 fish per tank). All diets are in triplicate except FSK4%, that is, in duplicate. n.s. stands for not significant. WW refers to a wet weight basis. ^1^Second polynomial model (quadratic), *Y* = 3.419*x*^2^ − 13.16*x* + 78.72. ^2^Simple linear regression, *Y* = −1.769*x* + 55.87. ^3^Simple linear regression, *Y* = − 1.724*x* + 50.90. ^4^Simple linear regression, *Y* = −2.264*x* + 21.95. ^5^Segmental linear regression, *Y*_1_ = −5.472*x* + 8.140, *Y*_2_ = −0.1356 (*x*− 1) + 2.668, *Y* = IF (*X* < 67, *Y*_1_, *Y*_2_), *X*_0_ = 67 (iodine in FSK1%).

## Data Availability

The data that support the findings of this study are available from the corresponding author upon reasonable request.

## Supplementary Materials

Supplementary Materials The supplementary materials that support the findings of this study can be found online. The growth performance and mineral composition of all-male population of Atlantic salmon postsmolt fed graded inclusion of fermented sugar kelp (FSK) are presented in supplementary materials for future reference in the use of the same fish when testing new raw materials. Figure S1A: comparing the mean and the trendline of final individual weight of all-male population and mixed population of Atlantic salmon postsmolts fed graded inclusion of fermented sugar kelp (FSK). *n* = 30 fish from all-male population, *n* = 165 fish from mixed population per diet, all diets are in triplicate except FSK4%, that is, in duplicate. Table S1A: growth performance indicators of all-male population of Atlantic salmon postsmolts, fed graded inclusion levels of fermented sugar kelp (FSK). Table S1B: whole-body and muscle proximate composition and mineral status of all-male population of Atlantic salmon postsmolt fed graded inclusion of fermented sugar kelp.
