# Supplementary material for: Growth Performance, Nutrient Digestibility, and Retention in Atlantic Salmon, Salmo salar L., Fed Diets with Fermented Sugar Kelp, Saccharina latissima
Source: Aquac Nutr. 2023 Nov 29;2023:6664947. doi: 10.1155/2023/6664947 (PMC10699978; doi:10.1155/2023/6664947)
Supplement: Supplementary Materials — The supplementary materials that support the findings of this study can be found online. The growth performance and mineral composition of all-male population of Atlantic salmon postsmolt fed graded inclusion of fermented sugar kelp (FSK) are presented in supplementary materials for future reference in the use of the same fish when testing new raw materials. Figure S1A: comparing the mean and the trendline of final individual weight of all-male population and mixed population of Atlantic salmon postsmolts fed graded inclusion of fermented sugar kelp (FSK). n = 30 fish from all-male population, n = 165 fish from mixed population per diet, all diets are in triplicate except FSK4%, that is, in duplicate. Table S1A: growth performance indicators of all-male population of Atlantic salmon postsmolts, fed graded inclusion levels of fermented sugar kelp (FSK). Table S1B: whole-body and muscle proximate composition and mineral status of all-male population of Atlantic salmon postsmolt fed graded inclusion of fermented sugar kelp. [file 6664947.f1.docx]

**Supplementary figures and tables**

**Figure A: Comparing the mean and the trendline of final individual weight of all-male population and mixed population of Atlantic salmon post-smolts fed graded inclusion of fermented sugar kelp (FSK).** n= 30 fish from all-male population, n= 165 fish from mixed population per diet, all diets are in triplicate except FSK4%, that is in duplicate.


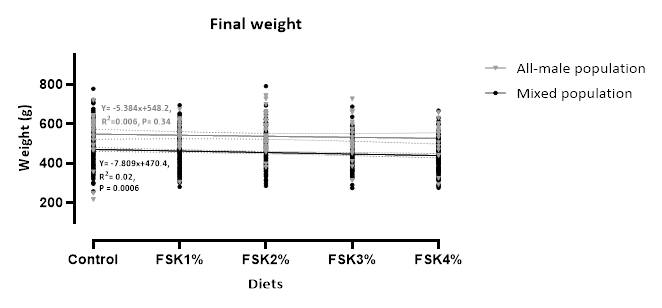


| **Table A: Growth performance indicators of all-male population of Atlantic salmon post smolts, fed graded inclusion levels of fermented sugar kelp (FSK).** | | | | | | | |
| --- | --- | --- | --- | --- | --- | --- | --- |
|  | **Control** | **FSK1%** | **FSK2%** | **FSK3%** | **FSK4%** | **Regression**  **(P-value, R^2^)** | **ANOVA** |
| IBW (g) | 216 ± 6 | 210 ± 4 | 221 ± 7 | 216 ± 7 | 215 ± 9 | n.s. | n.s. |
| FBW (g) | 544 ± 20 | 540 ± 16 | 556 ± 23 | 521 ± 30 | 526 ± 51 | n.s. | n.s. |
| WG (g) | 329 ± 15 | 327 ± 9 | 336 ± 10 | 305 ± 12 | 311 ± 15 | n.s. | n.s. |
| SGR (% day^-1^) | 1.30 ± 0.03 | 1.33 ± 0.02 | 1.32 ± 0.01 | 1.25 ± 0.03 | 1.26 ± 0.04 | n.s. | n.s. |
| K | 1.4 ± 0.02 | 1.2 ± 0.01 | 1.3 ± 0.01 | 1.3 ± 0.01 | 1.3 ± 0.05 | n.s. | n.s. |
| Notes: IBW = initial body weight (g), FBW = final body weight (g), WG = weight gain (g), SGR = specific growth rate, K = condition factor.  Data is presented as mean ± SEM (n=10 fish per tank, all diets are in triplicate except FSK4%, that is in duplicate).  n.s stands for not significant. | | | | | | | |

| **Table B: Whole-body and muscle proximate composition and mineral status of all-male population of Atlantic salmon post smolt fed graded inclusion of fermented sugar kelp.** | | | | | | | |
| --- | --- | --- | --- | --- | --- | --- | --- |
|  | **Control** | **FSK1%** | **FSK2%** | **FSK3%** | **FSK4%** | **T-test^1^**  **(P-value)** | |
| **Body composition (g 100g ^-1^ WW)** | | | |  |  |  |  |
| Protein | 17 ± 0.3 | - | - | - | 17 ± - | n.s. |  |
| Total fat | 14 ± 0.7 | - | - | - | 13 ± 0.6 | n.s. |  |
| Energy | 9387 ± 224 | - | - | - | 9020 ± 210 | n.s. |  |
| Ash | 2 ± 0.07 | - | - | - | 2 ± 0.01 | n.s. |  |
| Dry matter | 33 ± 0.6 | - | - | - | 32 ± 0.8 | n.s. |  |
| **Micro-mineral in whole-body (mg kg ^-1^ WW)** | | | |  |  |  |  |
| Mn | 2 ± 0.2 | - | - | - | 2 ± 0.4 | n.s. |  |
| Cu | 0.9 ± 0.02 | - | - | - | 0.7 ± 0.02 | P = 0.01 |  |
| Fe | 8 ± 0.1 | - | - | - | 11 ± 3 | n.s. |  |
| Se | 0.2 ± 0.008 | - | - | - | 0.2 ± 0.01 | n.s. |  |
| Zn | 26 ± 1.2 | - | - | - | 30±3 | n.s. |  |
| I | 0.3 ± 0.02 | - | - | - | 2 ± 0.05 | P <0.0001 |  |
| **Micro-mineral in muscle (mg kg ^-1^ WW)** | | | |  |  |  |  |
| Mn | 0.1 ± 0.02 | - | - | - | 0.2 ± 0.03 | n.s. |  |
| Cu | 0.3 ± 0.003 | - | - | - | 0.3 ± 0.005 | n.s. |  |
| Fe | 2 ± 0.03 | - | - | - | 2 ± - | n.s. |  |
| Se | 0.2 ± 0.006 | - | - | - | 0.2 ± 0.005 | n.s. |  |
| Zn | 4 ± 0.03 | - | - | - | 4 ± 0.2 | n.s. |  |
| I | 0.09 ± 0.004 | - | - | - | 0.7 ± 0.02 | P <0.0001 |  |
| Notes: Data is listed as mean ± SEM (n=5 fish per tank, pooled samples, all diets are in triplicate except FSK4%, that is in duplicate). n.s stands for not significant.  WW refers to a wet weight basis.  ^1^Two groups (control and FSK4%) of the all-male population were selected for the nutrient composition and mineral status analysis. | | | | | | | |
